# R.I.S.E. ID: Adaptations to the Current Political Climate for the Infectious Diseases Frontline

**DOI:** 10.1093/ofid/ofaf443

**Published:** 2025-07-25

**Authors:** Gonzalo Bearman, Priya Nori

**Affiliations:** Division of Infectious Diseases, Virginia Commonwealth University Health, Richmond, Virginia, USA; Division of Infectious Diseases, Department of Medicine, Montefiore Health System, Albert Einstein College of Medicine, Bronx, New York, USA

**Keywords:** burnout, engagement, leadership, management, professional development

## Abstract

Executive order–driven federal cuts and workforce contractions have immediately threatened health outcomes. These actions are broadly destructive, unfolding alongside ongoing, multistate outbreaks and deaths from vaccine-preventable illnesses. Furthermore, proposed steep cuts to Medicaid and the Affordable Care Act will have dire consequences for millions of Americans if passed into law. Given the urgency of collectively devastating federal actions, all healthcare providers must adapt, regardless of job role. Immediate societal harm through a loss of healthcare coverage and support of basic needs (eg, food stamps), erosion of pandemic preparedness, and loss of scientific expertise are just a few anticipated harms. Ultimately, increased downstream healthcare spending on a sicker society may ensue. While federal actions have “flooded the zone,” the infectious diseases workforce can actively R.I.S.E. (Recommit, Innovate, Speak Up, Evaluate) by recommitting to the most relevant issues impacting our patients, exercising our professional voices, and upholding our values.

## Teaser

Infectious Diseases has been continually challenged by social and political forces for decades (e.g., HIV, COVID, measles, etc.) but the current political climate requires intensified and coordinated efforts. The ID community can R.I.S.E (Recommit, Innovate, Speak Up, Evaluate), to address the most relevant issues impacting the health of our patients and communities.

If you don’t stick to your values when you’re being tested, they’re not values: they’re hobbies.

—Jon Stewart, American comedian

Since January 2025, executive orders have “flooded the zone” and negatively impacted the healthcare, public health, and scientific communities. Federal actions feel like a direct attack on our daily work and fundamental mission to create safer and healthier societies. We propose that the infectious diseases (ID) workforce actively R.I.S.E. (Recommit, Innovate, Speak Up, Evaluate) and uphold core functions through empowering actions that meet current challenges head-on (Table 1).

**Table 1.** R.I.S.E. Infectious Diseases Adaptations

Actions	Context	ID Provider Actions	Key Outcomes
Recommit	Funding cuts threaten the ID/public health workforce and ID patients (HIV, AMR surveillance, multistate vaccine preventable illness outbreaks); science and innovation will stall.	Reengage in patient care and education. Scrutinize federal actions impacting patients. Update clinical guidelines for urgent threats. Prepare for outbreaks. Promote ACIP-recommended vaccines among HCWs and vulnerable populations.	Decreased preventable deaths from infections. Vulnerable groups and ID workforce supported and protected.
Innovate	Current federal landscape is favorable for AI expansion into daily healthcare functions.	Embrace AI for patient care, research, and administrative tasks. Provide oversight and be mindful of ongoing risks.	Improved diagnostics and clinical care. Enhanced ID provider efficiency and satisfaction.
Speak Up!	Independent, expert opinions can shed light on what is at stake from harmful federal actions.	Share expert opinions to educate the public. Join advocacy activities. Communicate clearly and avoid jargon. Share persuasive patient stories.	Rebuilt trust in core public health functions like vaccination. Policies to maintain healthy societies, counteract misinformation, and protect patients upheld.
Evaluate	Career fulfillment vs productivity demands should be evaluated regularly.	Cut out low-value work. Protect key roles (education, mentoring, etc). Monitor changes to productivity targets. Know when to unplug.	Enhanced career satisfaction. Valuable time optimized. Workforce sustained.

Abbreviations: ACIP, Advisory Committee on Immunization Practices; AI, artificial intelligence; AMR, antimicrobial resistance; HCW, healthcare worker; HIV, human immunodeficiency virus; ID, infectious diseases.

## R: Recommit

Significant funding cuts paused groundbreaking work in communicable diseases for researchers in the United States (US) and abroad. Decades of global collaboration and gains in control of tuberculosis, human immunodeficiency virus (HIV), malaria, vaccine-preventable illnesses, and antimicrobial resistance are immediately threatened, and millions of preventable deaths may ensue [1–3]. Given these setbacks, ID providers should recommit to the foundational pillars of patient care, medical education, mentorship, and advocacy of pressing public health issues.

We should actively monitor federal actions impacting patients. For instance, on 22 May 2025, the US House of Representatives passed a budget reconciliation bill including significant reductions to funding through Medicaid and the Affordable Care Act (ACA) [4], compounding negative impacts of prior cuts across US Department of Health and Human Services (HHS) agencies (ie, National Institutes of Health, Agency for Healthcare Research and Quality, Centers for Diseases Control and Prevention [CDC], and Centers for Medicare and Medicaid Services) [5]. Key aspects of the House-approved bill include approximately $700 billion in Medicaid cuts over 10 years, stricter work and eligibility determinations, increased barriers to ACA access, cuts to states for Medicaid expansion, and states providing coverage to undocumented immigrants. As a result, nearly 14 million Americans could lose health insurance and skip or delay medical care, and hospitals may have to absorb costs due to unreimbursed care and worse health outcomes [6, 7]. Cuts to Ryan White funding and the AIDS Drug Assistance Program are conceivable [8, 9]. Considering 1 in 5 people in the US receive health and long-term care coverage through Medicaid, including 37 million children (Medicaid or Children's Health Insurance Program coverage), our most vulnerable ID patients could slip through the social safety net. Another executive order promising to lower drug costs for Americans may positively impact patients, but outcomes remain to be seen [10, 11].

We can first adapt by helping patients navigate increased bureaucratic hurdles to healthcare access. This requires an awareness of local support services like social workers, free clinics, and other community healthcare partners. Hospitals should update and disseminate policies to protect patient rights and ensure appropriate management of Immigration and Customs Enforcement interactions in healthcare settings. Unfortunately, undocumented patients with life-threatening conditions are foregoing medical care for fear of immigration enforcement in hospitals, clinics, and pharmacies, and some are dying outside the hospital. However, the healthcare community is actively pushing back by refusing searches without signed judicial warrants, refusing to turn over protected health information, reminding patients of their rights (in all languages) such as the right to remain silent and the right to legal counsel, and creating home visitation programs to deliver life-saving healthcare [12].

Likewise, maintaining a focus on high-risk, marginalized groups, we can improve our understanding of state reproductive healthcare laws and update local guidelines for maternal sepsis [13]. We should appreciate that tuberculosis is rising globally and understand how increased community transmission may impact patients and healthcare workers. We should adopt newer, evidence-based regimens for streamlined tuberculosis prevention and treatment [14, 15].

We should maximally prepare for an influx of vaccine-preventable illnesses like measles and pertussis with triage, isolation, testing, management, prevention, and contact tracing protocols in coordination with local health departments [16]. ID providers should appraise local community vaccination rates, and school immunization exemption laws by state, and maintain close communication with patients and families to enforce vaccine uptake [17].

We should actively adopt recent Advisory Committee on Immunization Practices (ACIP) recommendations for vaccination of healthcare workers, patients, and long-term care residents (eg, respiratory syncytial virus, influenza, meningococcal, pneumococcal vaccines), which may in turn prevent hospitalizations, excess antibiotic use, and resistance [18, 19]. However, subverting usual protocols, HHS now recommends coronavirus disease 2019 (COVID-19) vaccines for the highest-risk groups only, excluding healthy children and pregnant women. Thankfully, the CDC maintained COVID-19 vaccines on its pediatric schedule (with shared decision-making) and protected their availability through Vaccines for Children, but barriers to insurance coverage and access may ensue [20]. The American College of Obstetricians and Gynecologists and other organizations urge payors to continue insurance coverage since pregnancy is a high-risk condition and COVID-19 vaccines have proven safe and effective in pregnancy [21].

While tariffs are rapidly evolving, we should anticipate supply chain disruptions to personal protective equipment, pharmaceutical ingredients, etc, and stockpile equipment and antimicrobials used to disrupt outbreaks of transmissible illnesses.

Increased scrutiny of work visas means that institutions could face additional barriers to hiring and sustaining nonpermanent resident physicians for essential and already strained ID functions (eg, clinical ID, infection prevention, outpatient parenteral antimicrobial therapy). ID program directors and division chiefs should stay informed on work visa navigation and provide ongoing assistance [22].

Finally, repositories for important public health guidelines subject to political interference (eg, HIV preexposure prophylaxis, sexually transmitted infection management, age-appropriate vaccines) can be archived and maintained elsewhere (eg, professional society websites, clinical decision support tools) [23]. Professional society guidance and local ID expertise remain crucial for upholding safe healthcare in the absence of CDC advisory groups such as the Healthcare Infection Control Practices Advisory Committee (HICPAC) and ACIP.

## I: Innovate

While recent federal actions are mostly damaging, artificial intelligence (AI) is poised to expand under a favorable stance by the current Administration [24]. Expanded use of healthcare in AI could be an upside to an otherwise adverse political climate by enhancing clinical efficiency and guideline-concordant care and augmenting academic, patient safety, and leadership roles. For example, machine learning and AI promise to improve direct patient care through diagnostic aids, note-writing capabilities, prediction scores for drug resistance, and enhanced clinical decision support tools. Commercial electronic medical records already contain generative AI–embedded platforms [25–30]. Now is the time for clinical adoption while remaining cognizant of potential risks.

AI can streamline scholarly work through analytic and research platforms and improve administrative tasks like policy writing. As less time is spent on surveillance, data gathering, and data analysis, greater emphasis can be placed on critical decision-making, change implementation, and effective leadership of hospital programs [31, 32].

## S: Speak Up!

Our voices as ID specialists are critical. Opinions in both the academic and lay press should present concerns alongside evidence-based and action-oriented solutions to current dilemmas. Examples include opinion pieces on the dangers of defunding public health and the loss of the CDC's HICPAC [33, 34]. Additionally, ID specialists may advocate via viewpoints in professional society publications [23].

Other opportunities include participation in professional society advocacy activities and liaising with state medical societies and local legislators on pandemic preparedness, sustaining funding for HIV programs, and promoting vaccine uptake.

Gaslighting signifies deceit for one's own advantage, and the result is disengagement and erosion of confidence by the gaslit individual [35]. Be aware of gaslighting in medicine and commit to jargon-free communication with colleagues and patients. Hone the skills of persuasion and harness local patient stories with executives and lawmakers to help improve patient outcomes.

Gaslighting the public erodes the public trust. We should speak directly to communities and acknowledge the risks, benefits, and uncertainties of all aspects of healthcare. Trust is built and maintained by individualized discussions promoting evidence-based medicine in a style accessible to our patients.

## E: Evaluate

In challenging times, recognition of professional values is critically important for career fulfillment [36]. Evaluate critical functions and limit nonessential activities if hospital budgets are strained (eg, travel to meetings). Minimize low-value, uncompensated work that diverts attention from core functions (eg, dysfunctional hospital committees) or at least seek protection for the most meaningful, uncompensated roles like teaching and mentoring [36]. Anticipate a recalibration of clinical productivity targets at your institution and know what this means for you. Last, evaluate when your attention is under threat, you’ve had enough, and must unplug to focus on yourself and your family [37].

## CONCLUSIONS

The ID field has been shaped and continually challenged by social and political forces for decades (eg, HIV, COVID-19). Upholding our core patient care, public health, and patient safety mission is key to our empowerment and resilience. We can R.I.S.E. (Recommit, Innovate, Speak Up, Evaluate), redirect our attention and actions to only the most relevant issues impacting our patients and communities, and exercise our voices to safeguard our fundamental values.

## Notes

***Acknowledgments.*** The authors would like to acknowledge Ms Peggy Andrews-Depew for her invaluable assistance with final manuscript presentation.

***Disclaimer.*** The viewpoints expressed herein are exclusively those of the authors and do not represent those of their employers or professional societies.

***Potential conflicts of interest.*** Authors have no conflicts of interest to disclose.
